# Identifying Potential Sources of Phthalate Contamination in the Leaves of Stevia Rebaudiana (Bertoni) and the Development of Removal Technology

**DOI:** 10.3390/molecules29071627

**Published:** 2024-04-04

**Authors:** Mei-Li Xu, Yuanxin Cheng, Mo Feng, Qingguo Lu, Yunhe Lian

**Affiliations:** 1 Chenguang Biological Technology Group Co., Ltd., Handan 057250, China; meilixu@tju.edu.cn (M.-L.X.);; 2 Chenguang Biotech Group Handan Co., Ltd., Handan 056000, China; 3 Hebei Natural Pigment Technology Innovation Center, Handan 057250, China

**Keywords:** stevia rebaudiana, steviosides, phthalates

## Abstract

Steviosides extracted from the leaves of the plant Stevia rebaudiana are increasingly used in the food industry as natural low-calorie sweeteners. Phthalates in food are often assumed to arise from food containers or packaging materials. Here, experiments were carried out to identify the potential sources of DMP, DBP, DIBP, and DEHP in the leaves of stevioside through investigation of their content in native stevioside tissues, soils, and associated agronomic materials. The results show that phthalate contamination was present in all the samples tested, and the influence of regional factors at the provincial level on the content of plasticizers in stevia leaves was not significant. Phthalates in stevia leaves can be absorbed into the plant body through leaves and roots. Using resin removal, the phthalate content in stevioside glycosides was reduced to less than 0.05 ppm, and some indicators were far lower than the limit standard in EU food.

## 1. Introduction

Stevia rebaudiana belongs to a perennial herb of the Compositae family, native to Paraguay and Brazil in South America. In recent years, Stevia rebaudiana has attracted attention for its abundance of stevioside glycosides. Stevioside sweeteners have been used for several decades to add sweetness to various foods worldwide. However, the research on Stevia rebaudiana has mainly focused on the activity of its extraction [1], the sweet flavor characteristics and their improvement [2], and the application of stevioside glycosides [3]. There are few reports on the limitations of Stevia rebaudiana as a food additive.

Phthalates are a group of diesters of ortho-phthalic acid (dialkyl or alkyl aryl esters of 1,2-benzenedicarboxylic acid). Phthalates have a broad variety of uses. The typical application is for plastics, especially polyvinyl chloride (PVC). They are also utilized in the production of flooring and children’s toys and are incorporated into printing inks, perfumes, and nail polishes. According to 2021 data, the total global market for plasticizers was 8.4 million metric tons [4]. The widespread use of phthalates in various applications poses a significant challenge due to their extensive contamination of the environment. Phthalates are not chemically bonded within plastics, allowing them to be easily released into the external environment.

Studies reveal that high exposure to and the evident toxicity of phthalates affect human health and ecosystem functioning [5]. The primary concerns associated with phthalate exposure in humans and wildlife are their impact on reproductive systems, including fertility issues, developmental problems in newborns, and potential carcinogenic properties [6]. Phthalates exhibit toxicity towards various species ranging from algae, protozoa, mollusks, crustaceans, and fish to invertebrates [7].

Food containers or packaging materials are often assumed to be the source of phthalates in food [8]. Although steviosides are low-fat food additives, mg/kg levels of phthalates have been detected in their commercial products. Previous studies have revealed the production process and investigated the migration of phthalate in different plastic products [9]; however, in addition to the contamination during the product process, the stevioside leaf is another main source of phthalates. This study aimed to identify the content of the representative phthalates, i.e., dimethyl phthalate (DMP), di-dibutyl phthalate (DIBP), dibutyl phthalate (DBP), and diethylhexyl phthalate (DEHP), in different parts of Stevia rebaudiana, soils, and associated agronomic materials, and provide an insight into the source of phthalates in steviosides. It can provide theoretical support for the cultivation of Stevia. In order to ensure the product quality and safety of stevioside, the removal process of the plasticizer in stevioside was further developed, and the residual amount of plasticizer in stevioside was reduced to below the European food limit standard.

## 2. Results and Discussion

### 2.1. Confirmation of Phthalate Contamination in Stevioside Leaves

Identification of phthalates, DMP, DBP, DIBP, and DEHP, in raw Stevia rebaudiana Bertoni leaf samples was conducted using a GC-MS system. For the specific detection conditions, the reader can refer to our previous article [9]. The necessary validations were carried out. The confirmation criteria followed the European Decision 2002/657/CE [10].

The method for the determination of DMP, DBP, DIBP and DEHP in stevioside leaf was validated in terms of the linearity, precision, recovery, and limits of detection (LOD) and quantification (LOQ).

The results of Table 1 show the good linearity for DMP, DBP, DIBP, and DEHP with a correlation coefficient (R^2^) higher than 0.9994. From the data in Table 2 and Table 3, we can see that the three phthalates, DMP, DIBP, and DBP, exhibit good system stability, with an RSD below 5%. The RSD for the DEHP was 7%. In terms of the evaluation of the daytime precision, the RSDs of the four plasticizers were almost double the original values. To better control the product testing precision, the samples were tested on the same day that they were collected.

**Figure 1:**
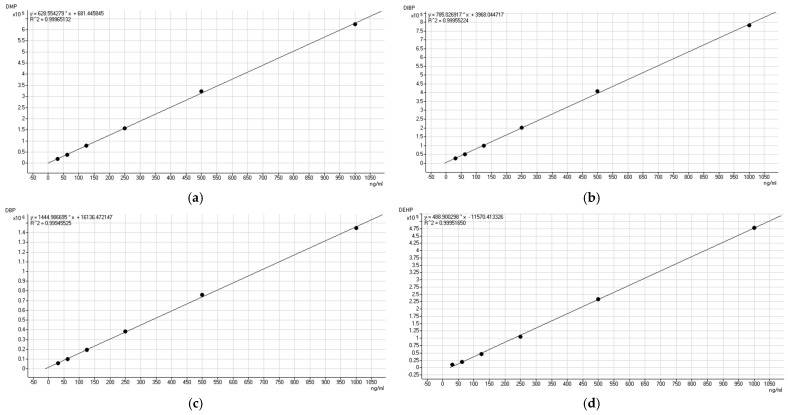
Standard working curves of four plasticizers, (**a**) DMP, (**b**) DIBP, (**c**) DBP and (**d**) DEHP.

**Figure 2:**
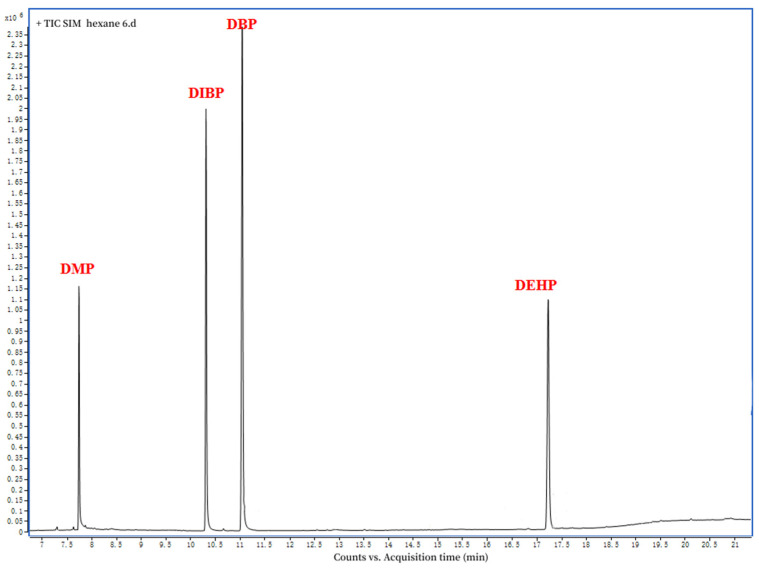
Mass spectrum of the standard sample of the phthalate mixture.

Figure 1 depicts the GC−MS chromatographic separation of DMP, DBP, DIBP, and DEHP in the standard solution (1000 mg kg^−1^) and the stevioside leaf sample. The retention times of the standard solution were 7.74 min for the DMP, 10.31 min for the DIBP, 11.05 min for the DBP, and 17.24 for the DEHP (Figure 2).

### 2.2. Potential Contamination Sources of Phthalates in Agriculture

In order to augment agricultural output, intensive agricultural practices, particularly plant harvesting, impose substantial pressure to utilize fertilizers and related soil amendments [11]. To evaluate the ecological repercussions of these applications and agricultural methods on DMP, DBP, DIBP, and DEHP contamination, we performed an analysis on diverse agronomic substances like pesticides, fertilizers, and mulching films, employed in the agricultural production procedure.

Table 4 reveals the presence of DMP, DBP, DIBP, and DEHP in pesticide, fertilizer, and mulching film. The concentrations of these phthalates in the mulching film were significantly higher than those in fertilizer and pesticide, with values hundreds or thousands of times greater.

The result of the phthalate concentrations in different agronomic materials supports the fact that vegetation can promote plasticizers levels in stevioside leaves. With intensive agricultural activities, mulching film is applied to boost the agricultural productivity and reduce the planting cost during the growth period of stevioside; thus, plasticizer levels in stevioside leaves can be elevated owing to the application of mulching film containing plasticizers. With the broad range of applications of plasticizers in industry, daily life, and manufacturing agronomic materials, there is serious pollution of the air, water, and soil, which results in phthalate contamination in food materials.

### 2.3. Distribution of Phthalates in Stevioside Plant Tissues

Table 1 demonstrates that the analytical method utilized for measuring the DMP, DBP, DIBP, and DEHP concentrations in soils and various plant tissues was suitable for accurately quantifying these substances even at low levels.

Quantitative and qualitative analysis of DMP, DBP, DIBP and DEHP were performed on seven groups of samples from seven native experimental fields. As seen from Table 5, all the stevioside leaf samples were contaminated by phthalates to different degrees. DMP, DBP, DIBP and DEHP were presented in all the stevioside leaves samples, with ranges of 0.015–0.384, 0.335–1.684, 0.208–1.437, and 0.092–1.136, respectively. The detection results indicate that phthalate contamination was present in all the samples tested, and the influence of regional factors at the provincial level on the content of plasticizers in stevia leaves was not significant. A similar conclusion was reached by Wang et al. in the research on the spatiotemporal dynamics of phthalates in tea plants growing in different geographical environments [12].

All the samples were collected by the researchers from the Stevia rebaudiana Bertoni planting base. During the transport and storage process, the samples were wrapped with paper bags; so, no phthalates migration occurred from the packaging materials during the preparation and analysis process. Therefore, it can be ensured that the DMP, DBP, DIBP, and DEHP already existed in the Stevia rebaudiana Bertoni before the sample was collected.

**Figure 3:**
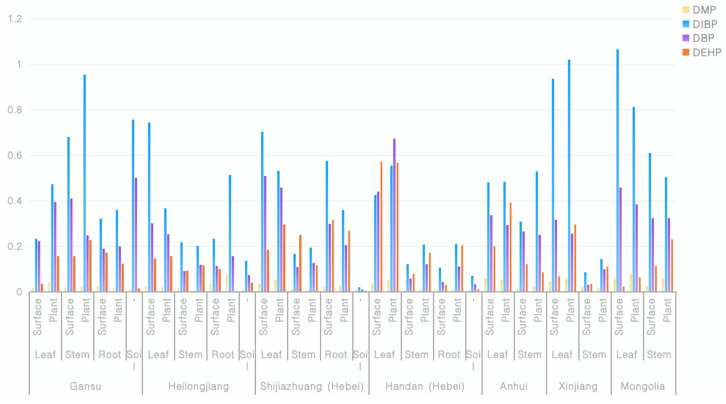
Concentration of plasticizers determined in Stevia rebaudiana Bertoni samples from different fields.

Table 5 demonstrated that the analytical method utilized for measuring DMP, DBP, DIBP, and DEHP concentrations in soils and various plant tissues is suitable for quantifying these substances accurately even at low levels. Thus, we proceeded with a comprehensive investigation involving 15 sets of soil and plant samples, including stems and roots. As shown in Figure 3, the DMP, DBP, DIBP and DEHP in plants had ranges of 0.0062–0.0584, 0.1184–0.3242, 0.1938–0.9538, and 0.0867–0.2303 in stems and 0.0080–0.0741, 0.1105–0.2040, 0.2102–0.5132, and 0.050–0.2677 in roots. The data indicated that the plasticizers accumulated in all tissues at different levels. While the mechanisms were not specifically investigated, we hypothesize that the distribution of phthalates among different organs of stevioside may be influenced by the lipophilic solid components and the transpiration stream flow rate of various parts of the plant [13].

From the data in Figure 3, we can see that the plasticizer composition in the leaves was generally high. It is inferred that air, dust, and other plant amendments, rather than the soil, were the main source of contamination of the plasticizers in stevioside leaves. According to the data from the experimental samples in Gansu, Heilongjiang, and Shijiazhuang, we can see that the patterns of plasticizer content on the plant surfaces and within the plant bodies were inconsistent. However, when taking the overall plasticizer content in the soil into consideration, we see that when the content of plasticizers in the soil was low, the plasticizer content on the plant surface was higher than that within the plant body; when the content of the plasticizers in the soil was high, the plasticizer content within the plant body exceeded that on the plant surface. Therefore, it is inferred that aside from the root absorption, plasticizers in stevia leaves can be absorbed into the plant body through the leaves. This conclusion is consistent with the results obtained by Professor Wang et al. that indicated a positive correlation between the plasticizers in tea leaves and the air quality [12]. The use of plastic mulch during the cultivation of stevia provides an environment in which the plasticizers in the mulch come into contact with stevia and are absorbed by the stevia leaves through dust or air during the growth period.

In conclusion, phthalate contamination was present in all the samples tested, and the influence of regional factors at the provincial level on the content of plasticizers in stevia leaves was not significant. Phthalates in stevia leaves can be absorbed into the plant body through the leaves and roots.

### 2.4. Plasticizer Removal Technology

Step 1 Preparation of stevioside glycoside solution. Take the stevioside solution, which has already had other impurities removed, mix it well, and test the plasticizer. Adjust the content of the stevioside liquid to a solid concentration of 10–13% (*m*/*v*) and an ethanol concentration of 5–10% (*v*/*v*), and set aside.

Step 2 Resin activation. The following procedure was used to activate the XDA-8G resin: ① Soak the resin in 1BV 95% ethanol solution for 2 h, and use water to replace the ethanol until the ethanol concentration of the effluent is less than 1%; ② Treat the resin with 3BV 4% NaOH aq. at 1.5BV/h flow rate, and replace with pure water to reach pH ≤ 9; ③ Treat the resin with 3BV 4% HCl aq. at 1.5BV/h flow rate, and replace with pure water until the effluent pH ≥ 4.

Step 3 Removal of plasticizer. Control the flow rate of the stevioside solution at 1 BV/h for resin treatment. After treating the material, mix the sample well, and test the plasticizer content.

Table 6 demonstrates that through the resin removal process, the phthalate content in the stevioside glycosides was reduced to less than 0.05 ppm, and some indicators were far lower than the limit standard for EU food.

## 3. Materials and Methods

### 3.1. Chemicals and Materials

A phthalates mixture containing DMP, DEP, DIBP, DBP, DMEP, DMPP, DEEP, DPP, DHXP, BBP, DBEP, DCHP, DEHP, Diphenyl phthalate, DNOP, and DNP standards in n-hexane at a concentration of 1000 mg/kg was obtained from o2si smart solutions (Charleston, SC, USA). A phthalates mixed standard solution was purchased from o2si smart solutions (Charleston, SC, USA), and it is a solution in n-hexane with a concentration of 1000 mg/kg. Hexane was purchased from Fisher Scientific (Waltham, MA, USA). XDA-8G resin was purchased from Xi’an Lanxiao Technology New Materials Co., Ltd. (Xi’an, China). The stevioside liquid was obtained from the stevioside production workshop of Chenguang Biotechnology Group Co., Ltd. (Handan, China).

### 3.2. Apparatus

Ultrapure water was prepared using a Master-S UVF model laboratory water purification system (Hetai Instrument Co., Ltd., Shanghai, China). The samples were triturated using an FM-100 universal grinder (Taisite Instrument Co., Ltd., Tianjin, China). The extraction efficiency was enhanced using an XW-80A model vortex instrument (Jingke Co., Ltd., Shanghai, China) and an Hs3120 model ultrasound cleaner (Tianjin Hengao Co., Ltd., Tianjin, China) operating at 600 W. Subsequently, the phase after extraction was separated using an H-2020R model freezing centrifuge (Changsha Xiangyi centrifuge Equipment Co., Ltd., Changsha, China).

### 3.3. Sample Collection and Preparation

Samples of Stevia rebaudiana Bertoni leaves, stems, roots, and soils containing root systems were randomly collected from seven different planting bases in China. The samples were obtained from farmers at the Handan planting base and included pesticide (spirodiclofen), fertilizer (potassium ammonium nitrate), and mulching film, which are listed in Table 7. The collected leaf, stem, root, and soil samples were allowed to air-dry naturally and then stored at room temperature, wrapped in white papers. until extraction and analysis.

### 3.4. Extraction Procedure

Extraction and analysis were conducted using a modified method of the PRC National Standard GB/T 21911-2008 [14].

A 10.0 g (leaves, stems and roots) aliquot of the samples was washed with ultrapure water, and the washed water was filtered through qualitative filter paper into a 2 mL vial. Then, 1 µL of this solution was injected into the GC-MS for analysis, and the results represented the phthalate content on the samples’ surface.

The samples were air-dried naturally, smashed, and transferred into 250 mL conical flasks. We added 100.0 mL n-hexane to the conical flasks, performed ultrasonic extraction for 40 min, and repeated this process with new solvent. We collected and mixed the extracting solution, metered the volume, centrifuged it at 5000 rpm for 5 min at 10 °C, and then dealt with the supernatants according to the above procedure; the result was the phthalate content of the samples.

The soil and fertilizer samples were both weighed at 10.0 g and the mulching film samples at 50.0 mg; we performed ultrasonic extraction twice with 50 mL, 50 mL, and 20 mL of n-hexane, respectively, and then, we dealt with the extraction solution, according to the above procedure.

### 3.5. Gas Chromatography–Mass Spectrometry (GC-MS)

Analytical measurements were performed using an Agilent Technology model 7000B mass spectrometer equipped with an electron impact (EI) detector, a 7693 series auto injector, and a 7890A model gas chromatograph. Separations were carried out on a DB-5MS fused-silica capillary column (30 m length × 0.25 mm internal diameter) coated with a 0.25 μm bonded film of 5% polyphenol methyl silicone.

The samples were analyzed using single ion monitoring (SIM) in the Agilent GC–MS system. A 1 μL sample was injected in splitless mode, with a helium carrier gas flow rate of 1.2 mL min^−1^. The temperatures for the front inlet, interface, MS source, and MS quad were set at 250, 280, 230, and 150 °C, respectively. Electron impact ionization mode was employed at 70 eV. The oven program consisted of a 1 min hold at 60 °C, followed by a ramp of 20 °C min^−1^ to 220 °C with a 1 min hold and, finally, a ramp of 5 °C min^−1^ to 265 °C.

### 3.6. Data Analysis

Quantitative and qualitative analyses were conducted using ion pair peaks in the chromatographic profiles of the samples, which were compared to standard chromatograms. Quantification was accomplished using the external standard method. The construction of a matrix-matched standard curve for Stevia rebaudiana Bertoni leaves involved fortifying blank samples with a 10-gradient MS analysis. Subsequently, the concentrations of DMP, DBP, DIBP, and DEHP were determined by comparing their respective external standards following replicate analysis.

## Figures and Tables

**Table 1:** Major analytical performance of the analytical method.

Analyte	Regression Equation	R^2^	LOD (mg kg^−1^)	LOQ (mg kg^−1^)	Recovery (%)
DMP	y = 628.55x + 681.44	0.9996	0.0002	0.0007	94.58
DIBP	y = 785.03x + 3968.04	0.9995	0.0090	0.0300	91.85
DBP	y = 1444.99x + 16136.47	0.9994	0.0250	0.0840	99.58
DEHP	488.90x − 11570.41	0.9995	0.0510	0.1700	94.77

x is the mass concentration (mg/kg), and y is the peak area. Recovery values at the concentration of the added standard (μg/kg).

**Table 2:** Instrument stability verification (ISV).

Sample	DMP	DIBP	DBP	DEHP
Standard Sample-1	358,172.3	518,311.4	552,384.8	224,958.4
Standard Sample-2	341,858	484,728.1	511,383.8	197,670.5
Standard Sample-3	340,606.1	479,139	507,285.1	202,576.8
Average	346,878.8	494,059.5	523,684.6	208,401.9
RSD (%)	2.8	4.3	4.8	7.0

**Table 3:** Daytime stability verification (DSV).

Time	DMP (mg kg^−1^)	DIBP (mg kg^−1^)	DBP (mg kg^−1^)	DEHP (mg kg^−1^)
21.05.2023 a.m.	0.227	1.019	1.539	0.857
21.05.2023 p.m.	0.208	1.135	1.673	0.849
22.05.2023	0.218	1.221	1.793	1.080
Average	0.218	1.125	1.668	0.929
RSD (%)	4.3	9.0	7.6	14.1

**Table 4:** Phthalate concentrations in different agronomic materials. All samples were prepared independently.

Materials	DMP	DIBP	DBP	DEHP
	(mg kg^−1^)			
Pesticide	0.015	0.005	0.046	0.029
Fertilizer	0.001	0.028	0.025	0.066
Mulching film	5.700	447.538	245.479	7.824

**Table 5:** Concentrations of DMP, DBP, DIBP and DEHP in stevioside leaf in seven native experimental fields. Concentrations are expressed on a dry matter basis.

Group NO.	Field	DMP	DIBP	DBP	DEHP
		(mg kg^−1^)			
1	Gansu	0.138	0.653	0.667	0.628
2	Heilongjiang	0.384	1.684	1.437	1.136
3	Shijiazhuang (Hebei)	0.015	0.925	0.886	0.559
4	Handan (Hebei)	0.053	0.538	0.474	0.176
5	Anhui	0.094	0.570	0.337	0.133
6	Xinjiang	0.212	0.335	0.208	0.139
7	Mongolia	0.163	0.504	0.325	0.092

**Table 6:** Data from plasticizer removal experiments.

Sample	DMP	DIBP	DBP	DEHP
	(mg kg^−1^)			
Original solution-liquid	0.0101	0.0430	0.0887	0.0680
Original solution-powder	0.1201	0.5158	1.0641	0.8159
After removal-liquid	0.0015	0.0040	0.0031	0.0036
After removal-powder	0.0179	0.0481	0.0368	0.0431

**Table 7:** Origin information of the samples.

**Sample**	**Anhui**	**Mongolia**	**Xinjiang**	**Heilongjiang**	**Gansu**	**Shijiazhuang**	**Handan**
Leaves	✔	✔	✔	✔	✔	✔	✔
Stems	✔	✔	✔	✔	✔	✔	✔
Roots				✔	✔	✔	✔
Soils				✔	✔	✔	✔
Pesticide							✔
Fertilizer							✔
Mulching film							✔

## Data Availability

Data are contained within the article.
